# A Multi‐City Assessment of Genomic Evolution in the Native Wildflower *Impatiens capensis*

**DOI:** 10.1111/eva.70218

**Published:** 2026-04-22

**Authors:** L. Ruth Rivkin, Colin J. Garroway, Marc T. J. Johnson

**Affiliations:** ^1^ Department of Biological Sciences University of Manitoba Winnipeg Manitoba Canada; ^2^ Polar Bears International Bozeman Montana USA; ^3^ Conservation Science Wildlife Health San Diego Zoo Wildlife Alliance Escondido California USA; ^4^ Department of Ecology and Evolutionary Biology University of Toronto Toronto Ontario Canada; ^5^ Department of Biology University of Toronto Mississauga Mississauga Ontario Canada

**Keywords:** effective population size, genetic diversity, jewelweed, local adaptation, urbanization

## Abstract

Urbanization is a major driver of environmental change that shapes the evolution of populations. However, the environmental differences amongst cities and their effects on neutral and adaptive evolution are less well understood. We investigated the contribution of city‐level variation to patterns of genetic evolution in *Impatiens capensis* , a native wildflower found in parks and green spaces in many cities across eastern North America. While the mixed mating system and flexible pollination requirements of *I. capensis* likely contribute to its resilience to urbanization, microenvironmental differences among cities may shape how this species evolves in cities. We used genotype‐by‐sequencing to evaluate genetic variation, contemporary demographic history, and genetic signatures of local adaptation in plants sampled from urban and rural sites across 10 cities in Ontario, Canada. Urbanization and city size shaped the amount of genetic diversity present at sites and were associated with fine‐scale spatial genetic structure. We identified a signal of repeated population bottlenecks across all cities, corresponding to the timing of rapid urban expansion in the region. City size was the environmental predictor most strongly associated with multilocus selection, highlighting the contribution of city variation to adaptive genomic evolution. Our findings provide one of the first examples of parallel demographic shifts in response to urbanization in plants and offer insights into why a native wildflower like *I. capensis* may be particularly resilient to urbanization. Taken together, our results emphasize the role that urban parks can play in maintaining genetic diversity and facilitating adaptation, suggesting that prioritizing greenspace conservation is critical for promoting urban biodiversity.

## 1 Introduction

Understanding population responses to human‐driven environmental change is essential for mitigating biodiversity loss. Changes in land use, such as urbanization, deforestation, and expansion of agricultural lands, are key drivers of recent extirpation and extinction events worldwide (IPBES 2019). Populations that are resilient to environmental change are important contributors to local and regional biodiversity. The amount of genetic variation in a population is a critical component of resiliency to stress and perturbations (Hughes et al. 2008). Populations with more genetic variation are more resilient to environmental change because the rate of adaptation scales directly with the amount of genetic variation in fitness (Fisher 1930; Barrett and Schluter 2008; Kardos et al. 2021). Genetic variation is in turn shaped by population size, habitat connectivity, and selection pressure. Thus, habitat loss or fragmentation may affect population persistence and lead to adaptive or maladaptive responses (Huxel and Hastings 1999; Frankham et al. 2019). Species responses to anthropogenic disturbance can enhance our understanding of how population resilience is maintained or lost in light of global change.

Urbanization causes significant environmental change. Cities are among the fastest growing environments in the world, with more than half of the global population currently living in urban areas (Ritchie et al. 2024). Given their ubiquitousness, cities can serve as replicated natural experiments, allowing for repeated tests of environmental change on ecological and evolutionary processes (Johnson and Munshi‐South 2017; Santangelo et al. 2020). Urban habitats share many characteristics, including impervious surfaces, high levels of disturbance, elevated temperatures, and managed greenspaces (McDonald et al. 2020). Additionally, cities that occur in close geographic proximity share regional climatic conditions. The urban environment can exert strong selection pressure and in some circumstances drive rapid evolutionary responses in urban species (Rivkin et al. 2019; Charmantier et al. 2024). For example, urban plants have evolved reduced seed dispersal in response to habitat fragmentation (e.g., Cheptou et al. 2008), mechanisms of increased antiherbivore defense (e.g., Santangelo et al. 2022), and increased tolerance to elevated temperatures (e.g., Woudstra et al. 2024). Urbanization also shapes patterns of genetic diversity and differentiation across many species due to changes in the strength of genetic drift and gene flow (Miles, Rivkin, et al. 2019; Schmidt et al. 2020).

Despite their general similarities, even nearby cities can differ widely in size, developmental histories, and socioeconomic factors, contributing to variation in urban ecological and evolutionary dynamics (Alberti et al. 2020; Des Roches et al. 2020). Large and rapidly growing cities can alter genetic variation and exert strong selection pressure on species due to the pace and magnitude of environmental change (Charmantier et al. 2024). Given these differences, studies that compare population genetic responses between nearby cities of different sizes and histories have the power to detect fine‐scale differences in evolutionary patterns that may go undetected when examining only a single city (Combs et al. 2018; Miles, Rivkin, et al. 2019; Schmidt et al. 2020; Fidino et al. 2021; Santangelo et al. 2022).

While many species are extirpated from cities due to habitat loss or stressful conditions, others persist and thrive. These species often exploit urban spaces for habitat, food resources, or dispersal mechanisms (McKinney 2002). Several well‐studied plant species (e.g., white clover [ *Trifolium repens* ], common dandelion [ *Taraxacum officinale* ], and sacred hawksbeard [*Crepis sancta*]) have evolved to grow in urban habitats, thriving in lawns, sidewalk cracks, and roadside verges (Cheptou et al. 2008; Larson et al. 2014; Santangelo et al. 2022). Other species thrive only in urban greenspaces or remnant natural habitats, such as parks or waterfronts. These species may have previously occupied larger areas before the city's expansion but are now restricted to the remaining suitable habitat within the city (Callaghan et al. 2019; Rivkin et al. 2019). Understanding the mechanisms that contribute to the persistence or extirpation of these species can provide useful information about how and when populations might be expected to survive in the face of rapid anthropogenically driven environmental change.

**FIGURE 1:**
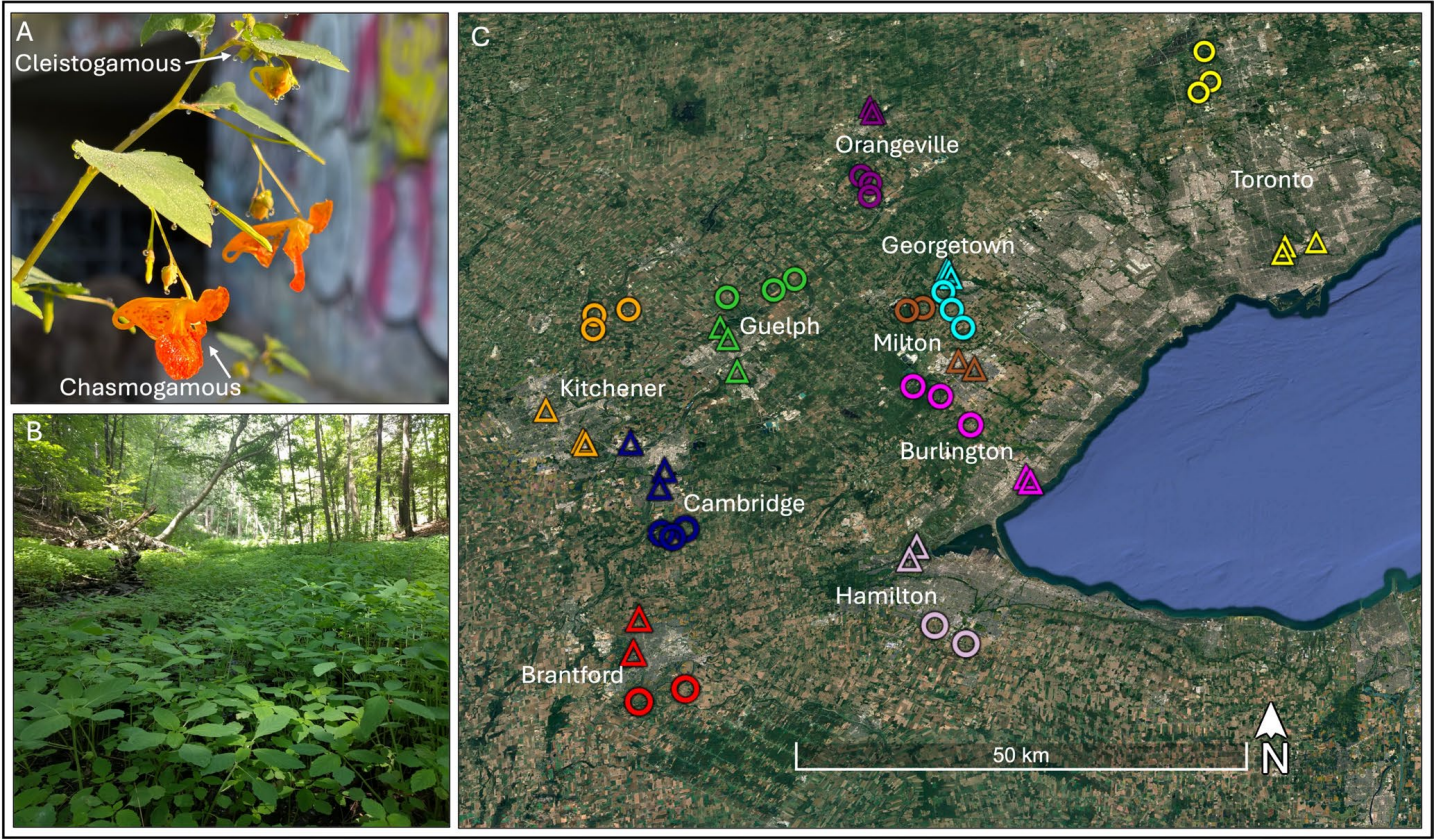
Depiction of study species and sites. (A) *Impatiens capensis* flowers, with the large, outcrossing chasmogamous and smaller, self‐fertilizing cleistogamous flowers highlighted. (B) Densely growing urban *I. capensis* population prior to flowering. (C) Map of sample sites, with each city labeled and denoted by a different color. Rural sites are displayed with circles, and urban sites with triangles. Photo credits: Ruth Rivkin, map credit: Google Earth Pro (2025).

We explored the role of environmental heterogeneity within and among cities on patterns of neutral and adaptive genetic variation in orange jewelweed, *Impatiens capensis* Meerb. (Balsaminaceae) populations. *I. capensis* is a wildflower native to eastern North America. Populations grow in wet, shady habitats. In cities, these habitats are found almost exclusively within parks and remnant natural habitats, where *I. capensis* often grows at high densities (Figure 1B). Urbanization was associated with declines in genetic variation in *I. capensis* populations in one large city, Toronto, Canada (Rivkin and Johnson 2022). Despite reductions in genetic diversity, pollination and outcrossing rates remained high for *I. capensis* in a different, more northern city (Ottawa, Canada; Barker and Sargent 2020). *I. capensis* has a mixed mating system, where each plant produces both self‐compatible, predominantly outcrossing (chasmogamous) flowers , and closed, obligately self‐fertilizing (cleistogamous) flowers (Figure 1A). Chasmogamous flowers are pollinated by a variety of species, but most commonly by bees and hummingbirds (e.g., *Bombus* spp., *Apis mellifera* and *Archilochus colubris* ; Schemske 1978). The mixed mating system and flexible pollinator requirements may contribute to the species resilience to urbanization by allowing persistence even if some pollinator populations decline in cities. However, urban resilience and patterns of evolution may also be shaped by microenvironmental differences among cities, a process that remains poorly understood. We addressed this knowledge gap by comparing genetic patterns across cities that are close enough together to share very similar climates but differ in their microenvironmental characteristics, sizes and densities, and developmental histories.

To better understand how differences and similarities among cities may shape evolutionary processes, we explored the emergence of population structure, demographic history, and genetic signatures of local adaptation in *I. capensis* . We greatly expanded on our previous study of *I. capensis* by exploring these processes across 10 cities located within 100 km of Toronto (Figure 1C). These cities varied in potentially important characteristics that could shape the ecology and evolution of *I. capensis* , including human population growth rate, city size, and developmental history. We asked three questions: (1) Do city characteristics and urbanization shape patterns of genetic diversity and genetic structure among *I. capensis* populations? (2) What is the demographic history of *I. capensis* across cities? And (3) Do environmental differences within and between cities contribute to signatures of selection consistent with genomic patterns of local adaptation in *I. capensis* populations? Our results shed light on the contributions of cities to the evolution of a native wildflower, including identifying possible avenues for population resilience to large‐scale environmental change.

## 2 Materials and Methods

### 2.1 Sampling Protocol

*Impatiens capensis* is an annual species that grows in damp habitats, such as in forest depressions, ditches, and along the margins of marshes, ponds, lakes, and rivers. In ideal growing conditions, plants can reach 2 m, and population size can number in the thousands (Figure 1B). In September 2019, we sampled 53 *I. capensis* populations growing in urban and rural sites across 10 cities in southern Ontario (Figure 1C). Each city has undergone a unique developmental history and their boundaries remain largely distinct from one another, although recent urban sprawl has connected the suburbs of some cities at their margins (e.g., Burlington and Hamilton, Kitchener and Cambridge). *I. capensis* is native to the region, but due to agricultural and urban development, the species primarily persists in small pockets of natural habitat. Urban sites were sampled within the city borders and were typically located in parks. Rural sites were sampled outside city borders and were frequently found growing beside rivers or in shaded farmland. We collected 10–15 seeds from five to eight individuals at each site that were spaced at least 2 m apart. Immediately after collection, we placed the seeds in a cooler with ice packs and transferred the seeds to a −20°C freezer at the end of each day until DNA extraction could be performed.

### 2.2 Environment Quantification

To investigate the impact of city size on *I. capensis* population genetic evolution, we calculated the area (km^2^), human population density (number of people per km^2^), and population growth rate between 2016 and 2021 for each city (Table S1). We obtained all estimates of city size from the Government of Canada Census of Population dataset (Statistics Canada 2023).

We quantified the environmental conditions at each site to investigate how environmental heterogeneity influences genetic variation in *I. capensis*. We extracted the percent impervious surface area surrounding each site from the 2010 Global Man‐made Impervious Surface dataset v1 at a spatial resolution of 30 m (de Brown Colstoun et al. 2017). This dataset bins pixels into urban and non‐urban categories and uses surface reflectance to estimate the extent of the area within an urban pixel covered by impervious surfaces (de Brown Colstoun et al. 2017). We measured the vegetation surrounding each site using the Landsat Normalized Difference Vegetation Index (NDVI) from the United States Geological Survey AppEARS database (Didan 2021). We averaged monthly NDVI raster maps from May to September 2019, at a spatial scale of 1 km to obtain a mean growing season NDVI value for our sampling year. Lastly, we estimated climatic variation between populations using mean annual temperature and mean annual precipitation at each site from the Worldclim2 database at a spatial scale of 1 km (Fick and Hijmans 2017).

We compiled the separate environmental raster files into a single raster with four layers. We resampled the NDVI and climate layers to match the resolution and extent of the impervious surface area layer with the *projectRaster* function from the terra v1.7‐71 package (Hijmans 2024) in R v4.4.1 (R Development Core Team 2008). We evaluated differences in environment across the study area using a Principal Component Analysis (PCA). Lastly, we extracted the mean value from each layer at each site using a 20 m buffer radius (buffer area = 1256 m^2^).

### 2.3 DNA Extraction and Sequencing

Sequencing was performed in two batches because of delays caused by the COVID‐19 pandemic. The first batch was sequenced in 2020 and included samples from Toronto. The results for this batch are presented in Rivkin and Johnson (2022). We sequenced the remaining samples in 2021, following the same DNA extraction and sequencing protocol (Rivkin and Johnson 2022). Prior to extraction, we removed the seed coat from the seeds to prevent contamination from maternal DNA. We extracted DNA from one seed from each plant using the DNeasy Plant Mini Kit in tube format from QIAGEN (Germantown, MD, USA) and assessed the quantity of DNA from each extraction using a Qubit Fluorometer dsDNA High Sensitivity Assay (Thermo Fisher Scientific, Waltham, MA, USA). Library preparation and sequencing were performed by The Elshire Group (Palmerston North, New Zealand) using genotype‐by‐sequencing (Elshire et al. 2011). Samples were digested with the restriction enzyme ApeKI, tagged with combinatorial barcodes, and multiplexed into a single library. The samples were sequenced on an Illumina HiSeq XTen machine (Illumina Inc., San Diego, CA, USA) with 150 bp paired end reads.

### 2.4 Variant Discovery and Filtering

To avoid batch effects, we processed reads from all samples, including Toronto, in a single pipeline. We demultiplexed the raw sequences using Axe v0.3.3 (Murray and Borevitz 2018), trimmed adaptor and reverse barcode sequences with the custom batch_trim.pl script (https://github.com/relshire/GBS‐PreProcess), removing reads with a Phred score less than 20. We inspected the quality of the reads with fastQC v0.11.4 (Andrews 2015). We used BWA v0.7.12 (Li and Durbin 2009) to map reads to the scaffold level using the *I. capensis* reference genome (Schoen and Speed 2024), then aligned and sorted reads with samtools v1.17 (Li et al. 2009). We removed duplicate PCR and technical reads with Picard Tools v2.27.4 (Broad Institute 2019).

We jointly called SNPs using bcftools v1.17 (Li et al. 2009) with a maximum per sample depth of 50×. We then used bcftools *filter* to remove reads with low quality scores, strand bias, and read mismatch, multi‐allelic calls, and retain reads that occurred in at least one sample with a depth > 10× and genotype quality > 30. As a final step, we used VCFtools v0.1.17 (Danecek et al. 2011) to remove indels and variants with call rates < 80% in all samples and minor allele frequency < 0.01. After filtering, we retained 9021 high quality SNPs called across nine scaffolds from 228 individuals, with an average variant depth of 6.5×.

Lastly, we examined the data for batch effects between sequencing runs. We used a PCA of genetic variation to confirm that samples did not cluster based on sequencing run. Samples from two sites from the first batch formed distinct clusters from the rest of the samples. This pattern was also detected in Rivkin and Johnson (2022); thus we believe this to be a signal of true population differentiation and not batch effects.

### 2.5 Genetic Diversity

We first assessed patterns of genetic diversity among sample sites. We used pixy v1.2.10.beta2 (Korunes and Samuk 2021) to calculate nucleotide diversity (*π*) for each site using both variant and invariant sites. We calculated observed heterozygosity (*H*_O_) across variant sites with the *gl.report.heterozygosity* function from the dartR R package v2.9.7 (Gruber et al. 2018). Lastly, we calculated the number of private alleles (PA; i.e., alleles that occurred in only a single population) per site using gl.report.pa function from the dartR package. For both H_O_ and PA we used PLINK v1.90 (Chang et al. 2015) to filter SNPs for Hardy‐Weiberg Equilibrium (HWE; *p* < 0.001) to include only neutrally evolving loci. We also filtered SNPs for linkage disequilibrium in 10 kb windows (‐‐*indep‐pairwise 10 1 0.1*), retaining a total 3261 SNPs.

We tested for differences in genetic variation across habitats and cities. We first compared *π*, *H*_O_, and PA between urban and rural habitats using a linear mixed effect model with city included as a random effect. We next modeled changes in *π*, *H*_O_, and PA per site associated with impervious surface area, NDVI, mean annual temperature, mean annual precipitation, log‐transformed city area, and city growth rate. We did not condition our models on city or site to enable them to assess population‐level differences in genetic diversity attributable to variation in environment. Our sample sites were farther apart than the typical seed dispersal distance of this species (e.g., within 2 m; Schmitt et al. 1985), ensuring that our samples come from independent local populations and meeting the statistical assumptions of independence (Szulkin et al. 2020). We confirmed that the residuals were not spatially autocorrelated using a Durbin–Watson test (Cliff and Ord 1972) and assessed the assumptions of the models by plotting residuals and examining collinearity between predictors.

### 2.6 Population Structure

We quantified population genetic structure to identify the potential role of environment on relatedness among individuals. We first filtered the dataset for linkage disequilibrium and HWE to remove library artifacts and correct for non‐independence of linked SNPs (Pearman et al. 2022). We assessed pairwise genetic differentiation (*F*_ST_) among cities using the *stammpFst* function in the StaMMP v1.6.3 R package (Pembleton et al. 2013). We bootstrapped across loci 1000 times to generate 95% confidence intervals for each pairwise *F*_ST_ value to determine if cities were significantly differentiated from one another. We also investigated the inbreeding coefficient (*F*_IS_) for each site with the *gl.report.heterozygosity* function from the dartR package.

We evaluated genetic structure among individuals using a PCA generated with the *‐‐pca* flag from PLINK. In addition, we estimated individual ancestry coefficients using sparse nonnegative matrix factorization (sNMF; Frichot et al. 2014), implemented with the *snmf* function from the R package LEA v3.12.2 (Frichot and François 2015). sNMF takes a non‐model based approach to estimate individual admixture coefficients from multilocus genotype data comparable to those from STRUCTURE or ADMIXTURE (Frichot et al. 2014). We compared the fit of models with *K* = 1–15 clusters across 10 independent runs and selected the value of K with the lowest cross‐entropy score as the best‐fitting model (Frichot et al. 2014).

We next evaluated fine‐scale spatial genetic structure using a Moran's Eigenvector mapping (MEM) based analysis. We generated a genetic distance matrix for all samples using the *‐‐distance‐matrix* flag from PLINK, which calculates pairwise Identity‐by‐State distances for all pairs of individuals (Chang et al. 2015). We then used the R package MEMGENE v1.0.2 (Galpern et al. 2014) to evaluate genetic relatedness as a function of spatial distance between sites. We used the *mgQuick* function with default parameters to calculate the Euclidean distances between shared alleles across individuals and extracted the first three MEMGENE variables (i.e., the eigenvectors from a PCA of the fitted values from a redundancy analysis of genetic distances). We visualized the variables by mapping them onto the geographic coordinates for each sample.

### 2.7 Demographic History

We investigated how urbanization has shaped the demographic history of *I. capensis* across cities. Very recent changes in *N*_*e*_ can be estimated by considering the contribution of each generation to linkage disequilibrium among pairs of loci, allowing for the estimation of *N*_*e*_ within 200 generations (Novo et al. 2023). We used GONE (Santiago et al. 2020) to obtain historical estimates of *N*_*e*_ over the past 200 generations, with the highest resolution for the last 100 generations. This timeframe should capture the developmental history of each city (Table S1) because *I. capensis* is an annual with minimal seed bank persistence (Antlfinger 1989). Because the recombination rate of *I. capensis* is unknown, we ran GONE with a constant recombination rate of 1 cM/Mb for the whole genome. We accounted for variation in recombination rates across the genome due to partial self‐fertilization by removing loci pairs with elevated recombination rates (hc = 0.01). This approach provides an accurate estimate of linkage disequilibrium generated from genetic drift in both the recent and distant past (Santiago et al. 2020). Our results suggested extensive recent gene flow between cities, so we also ran GONE on all samples combined, treating them as a single population to identify demographic events across the entire region, as opposed to each city independently. We repeated this analysis 100 times to generate 90% confidence intervals surrounding the estimates of *N*_*e*_.

We also estimated contemporary *N*_*e*_ of the parental generation for each city using NeEstimator v2 (Do et al. 2014) implemented through a wrapper function (gl.LDNe) in the dartR.popgen v1.0.0 R package (Gruber et al. 2018). We performed a Waples correction based on the number of chromosomes in the *I. capensis* genome (*N* = 20) to improve the accuracy of the estimates (Gruber et al. 2018).

### 2.8 Genotype‐by‐Environment Association Tests

We tested for signatures of selection consistent with local adaptation using three complementary approaches. For all approaches, we filtered for linkage disequilibrium, retaining 3965 SNPs. We first conducted a Redundancy Analysis (RDA) to identify putatively adaptive loci associated with environmental variation among populations (Rao 1964). RDA is a constrained ordination method that uses multivariate regression to identify loci that are linearly correlated with different components of the environment (Capblancq et al. 2018). Importantly, RDA can identify both the loci most strongly associated with environmental variables and the selective gradients associated with combined environmental variables (Capblancq et al. 2018). This approach allows for putatively adaptive variation to be identified in complex environments rather than across a single gradient.

We implemented the RDA using the *rda* function from the vegan v2.6‐4 R package (Oksanen et al. 2008) including the same environmental predictors as in the genetic diversity analysis. Prior to running the RDA, we imputed missing SNPs with the *impute* function from the LEA package with the most likely genotype value computed from the genotype matrix. We identified adaptive loci following Forester et al. (2018). We fit a partial RDA with all environmental variables and conditioned the model on the first Principal Component (PC1) from the PCA of genetic distances to control for neutrally‐evolving population structure. We checked the model for multicollinearity (Variance Inflation Factor ≤ 3) and identified significant constrained axes after 999 permutations (*p* < 0.05). We selected candidate adaptive loci from SNPs on the significant axes with loadings greater than ±3 standard deviations away from the mean, which is equivalent to a 1% false discovery rate (FDR). Finally, we identified the environmental variable most strongly correlated with each candidate locus.

We also conducted genome scans for outlier loci using sNMF and pcadapt. The sNMF method detects outlier loci that may be under selection using *F*_ST_ values that account for underlying population structure (Martins et al. 2016). We computed *p*‐values for each locus with *K* = 6 clusters (the best fitting model identified by sNMF) using the *snmf.pvalues* function from the LEA package. We applied an FDR of 1% when selecting significant outliers to minimize the likelihood of false positives (Benjamini and Hochberg 1995). Lastly, we took a PCA‐based approach to detect outlier loci using the R package pcadapt v4.3.5 (Luu et al. 2017). This method handles the presence of admixed individuals well and assumes that loci that are correlated with population structure above a specified level are indicators of local adaptation (Luu et al. 2017). We specified an FDR of 1% and *K* = 3 clusters based on the PCA plotting results from pcadapt.

We considered outlier loci that were identified by all three detection scans to be robust candidates for natural selection. To evaluate the functional role of the shared loci, we investigated whether outlier loci overlapped known genes within 500 bp upstream and downstream of the reference genome. We identified regions using BEDTools v2.31.2 (Quinlan and Hall 2010), then queried sequences with BLASTx searches (Altschul et al. 1997), identifying up to 100 matches per region with a cutoff *e*‐value = 0.0001.

## 3 Results

### 3.1 Environmental Variation

The environmental PCA revealed patterns of environmental variation within and among cities. PC1 explained 57% of the variation in environments across the region and corresponded to increased impervious surface area and temperature, and decreased NDVI and precipitation, explaining broad differences between urban and rural environments (Figure S1A). Variation in precipitation was less than 200 mm across the entire region, whereas mean annual temperature varied by 4°C. PC2 accounted for 21% of the regional variation, and corresponded to increased impervious surface area, NDVI, and precipitation, and decreased temperature. This variation is best explained by fine‐scale heterogeneity likely due to the presence of urban parks and greenspaces (Figure S1B).

### 3.2 Genetic Diversity

**FIGURE 2:**
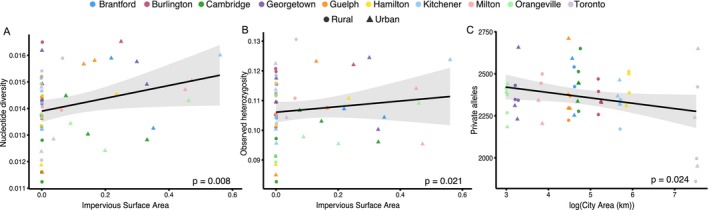
Genetic variation associated with urbanization and city size for 53 *Impatiens capensis* sample sites. (A) Nucleotide diversity (*π*) and (B) observed heterozygosity (*H*_O_) increased with the percentage of impervious surface area surrounding a site. (C) The number of private alleles (PA) per site declined with city size. City size has been log transformed. Shapes denote urban (triangle) and rural (circle) sites. Points have been jittered to better display variation among sites.

We identified clear effects of environmental variation in habitat and city size on genetic diversity (Figure 2; Table S2). Average *π* across all plants was 0.014 (range = 0.011–0.017) and *H*_O_ was 0.108 (range = 0.083–0.131). The average number of unique genetic variants (i.e., private alleles [PA]) among sites was 2327 (range = 1860–2170). There were no detectable broadscale effects of urban vs. rural habitat on genetic variation (*p* > 0.4 for all three models). However, *π* and *H*_O_ increased with impervious surface area (*π*: *R* ^2^ = 0.08, *p* = 0.008; *H*_O_: *R* ^2^ = 0.02, *p* = 0.021; Table S2), suggesting that genetic variation increased in habitats with more impervious surface coverage (Figure 2A,B). In contrast, PA decreased with city area (*R* ^2^ = −0.07, *p* = 0.024), suggesting that sites in large cities contain fewer unique alleles than sites in small cities (Figure 2C). Lastly, PA increased with temperature (*R* ^2^ = 0.02, *p* = 0.008, Table S2) and precipitation (*R* ^2^ = 0.05, *p* = 0.048, Table S2), suggesting that sites in warmer and wetter habitats were more genetically unique than sites in other environments.

### 3.3 Population Structure

Pairwise *F*_ST_ between cities was low, varying between 0.01 and 0.04, although all comparisons were statistically significant (Table S3). All estimates of *F*_IS_ were negative (mean = −0.136, range = −0.272 to −0.018; Table S1), suggesting that there was an excess of heterozygotes within populations.

**FIGURE 3:**
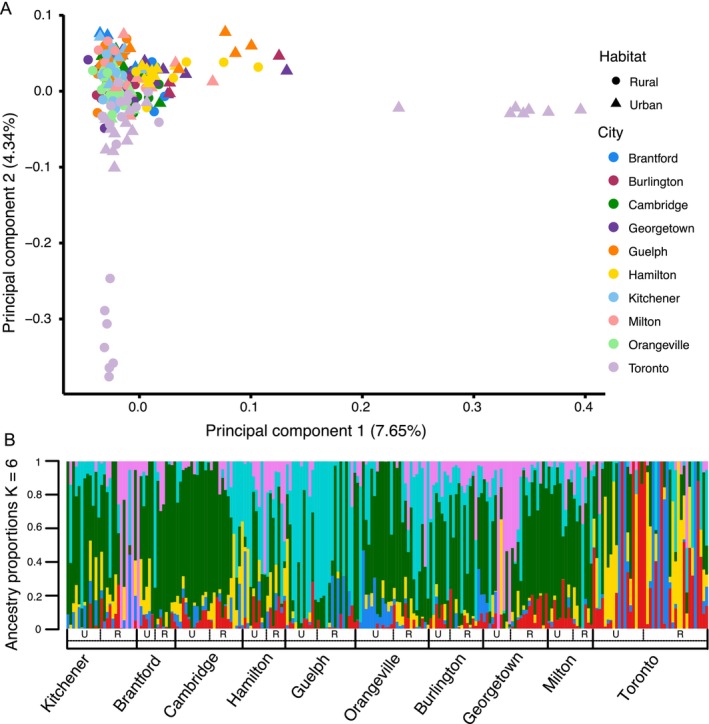
(A) Principal Component Analysis (PCA) of genetic structure of *Impatiens capensis* sampled from southern Ontario. Samples are color coded by city, with circles denoting rural sites and triangles denoting urban sites. The percent of variation explained by each PC axis is included in parentheses. (B) Ancestry proportions for *K* = 6 clusters generated by the best fitting sNMF model. Individuals are grouped by city ordered from west to east, with urban and rural sites denoted with a U or R, respectively. The colors represent shared genetic ancestry.

Cities did not form discrete genetic clusters and instead showed evidence of admixture. The PCA revealed three genetic clusters associated with the first two PC axes (Figure 3A). PC1 explained 7.65% of the genetic variation among samples, and while most samples clustered together, one urban site from Toronto separated out as a distinct cluster along the axis. PC2 explained 4.34% of the genetic variation, with one rural site from Toronto forming a distinct cluster. The sNMF analysis showed a similar degree of admixture (Figure 3B). The ancestry model fit plateaued at *K* = 6 clusters (Figure S2), with no single city standing out as genetically unique, although certain ancestral haplotypes were more common in specific cities (e.g., aqua in Guelph, and yellow and red in Toronto).

We identified broad and finescale signals of spatial genetic structure among individuals. The overall MEMGENE model explained 2.9% of genetic variation across all individuals. Within the model, the first three MEMGENE variables explained 38.1%, 22.8%, and 21.5% of shared genetic variation between individuals. Visualization of the MEMGENE eigenvectors identified different components of spatial genetic structure (Figure S3). The first variable (MEMGENE‐1) described variation between western and eastern cities, while MEMGENE‐2 identified spatial variation between the southern and northern cities. MEMGENE‐3 identified within‐city genetic variation, with some urban and rural habitats differing in genetic similarity.

### 3.4 Demographic History

**FIGURE 4:**
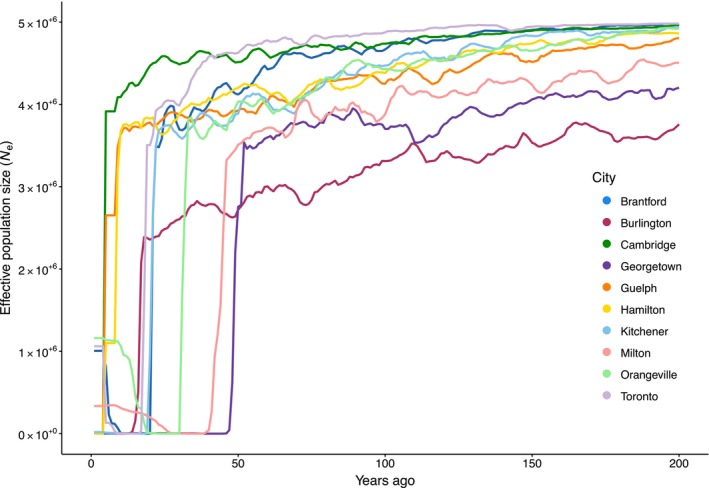
Recent demographic history of *Impatiens capensis* within 10 cities in the last 200 years (1 year = 1 generation) estimated with GONE. The *y*‐axis lowest value is *N*_*e*_ = 39 in Hamilton.

Contemporary *N*_*e*_ estimated by NeEstimator was low for all cities (Table S1). The average *N*_*e*_ across all cities was 92 plants, with the lowest in Toronto (*N*_*e*_ = 33) and the highest in Cambridge (*N*_*e*_ = 182). The reconstruction of recent demographic history by GONE identified a steep decline in *N*_*e*_ within the last 50 years, consistent with a population bottleneck in each city (Figure 4). Prior to the bottlenecks, *N*_*e*_ ranged from 2 to 5 million plants across all cities. Following the bottlenecks, contemporary *N*_*e*_ dropped below 100 plants in all 10 cities. Four cities showed some recovery of *N*_*e*_ following the bottleneck: Toronto, Milton, Brantford, and Orangeville, with *N*_*e*_ ranging from 300,000 to 1 million plants (Figure 4). When we analyzed all samples as a single population, we identified a similar bottleneck event approximately 35 years ago (Figure S4), suggesting that this trend is consistent across the region.

### 3.5 Genotype‐by‐Environment Association Tests

**FIGURE 5:**
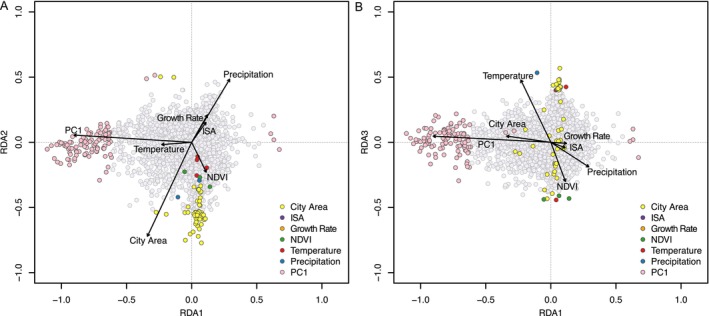
Ordination plots of (A) RDA1 and RDA2 and (B) RDA1 and RDA3 showing the association between candidate SNPs and environment. Impervious surface area is abbreviated to ISA. SNPs are color coded by their most highly correlated environmental predictor. Neutral SNPs are shown in light gray, and the vectors denote the environmental predictors. Both plots are scaled symmetrically by the square root of the eigenvalues. PC1 is the first principal component axis included from the genetic structure PCA to account for genetic structure in the model. Growth rate refers to the growth in human population size of each city over a 5‐year period.

The RDA identified 175 putatively adaptive loci associated with genetic structure (PC1), city size (i.e., city area and growth rate) and habitat heterogeneity (i.e., NDVI, impervious surface area, temperature, and precipitation). The RDA explained 3% of genetic variation across loci, suggesting that most SNPs in our dataset are evolving neutrally. The first three RDA axes were significantly associated with genetic variation: explaining 42%, 22%, and 8% of the variation, respectively. Toronto samples formed a distinct group from the other cities across all three RDA axes (Figure S5). These differences were most strongly associated with population structure and city size (Figure 5A). RDA3 was also associated with temperature, precipitation, and NDVI (Figure 5B). Population structure was the strongest contributor to multilocus selection, with 112 outlier loci most strongly correlated with PC1. City area was most strongly correlated with 50 outlier loci, while the remaining outliers were associated with temperature (4), NDVI (3), and precipitation (2).

The sNMF *F*_ST_‐based method identified 459 outlier SNPs, and the pcadapt method identified 126 outliers. Together, these methods identified 117 shared outlier loci. The RDA identified 130 SNPs shared with sNMF, and 13 shared SNPs with pcadapt. In total, 13 loci were identified as outliers across all three methods (Table S4). Of these, two loci were associated with city area and the rest with PC1. The BLASTx query of the 13 consensus outlier loci flagged 301 hits, 81 (27%) of which were associated with city area (Table S4). Most hits were uncharacterized proteins, although several of the outliers associated with city area were related to defensive functions (extensin and mucin‐like proteins).

## 4 Discussion

We identified clear associations between urbanization and patterns of genomic evolution in *I. capensis* . City area and impervious surface area were correlated with the amount of genetic diversity present at sites and contributed to finescale spatial genetic structure. We identified a signal of repeated demographic shifts across all cities that corresponded to the timing of rapid urban expansion in the region. Despite the evidence of recent genetic bottlenecks, contemporary genetic diversity and gene flow were high across the area. After accounting for population structure, city size was the only environmental driver of multilocus selection among sites, highlighting the role of cities in shaping the adaptive evolution of populations. Together, our results highlight the resilience of *I. capensis* to rapid environmental change and provide one of the first examples of parallel demographic change in response to urbanization in plants.

### 4.1 Genetic Diversity

City area, impervious surface area, and climate shaped the amount of genetic diversity present in populations and contributed to finescale spatial genetic structure. Increased impervious surface area was associated with increases in nucleotide diversity and heterozygosity (Figure 2). Additionally, sites with warmer annual temperatures, a variable that was positively associated with impervious surface area (Figure S1), harbored a greater number of unique genetic variants. These patterns were modest but consistent across populations. This pattern of increased genetic diversity associated with impervious surface area and temperature is most likely explained by urban *I. capensis* populations growing in high‐quality habitats with more consistent water availability, such as parks containing ponds and rivers. Rural populations, in contrast, were often found growing in roadside ditches with minimal canopy cover. This habitat is prone to drying out and is poorly suited for *I. capensis* growth and survival relative to the more stable habitats found in cities (Simpson et al. 1985). Inconsistent water availability can lead to seasonal population crashes, which may trigger genetic bottlenecks as plants die‐off during suboptimal growing conditions, reducing genetic variation in the population (Bouzat 2010). Given that genetic variation was high across all sites, gene flow across the region may have facilitated the rapid recovery of genetic variation in populations that experienced a crash (Bell et al. 2019). Indeed, the recent recovery of *N*_*e*_ in several cities supports this view. This result suggests that urban sites may act as genetic reservoirs for the more ephemeral rural sites.

Interestingly, we found different patterns of genetic variation when we compared the effects of urbanization across 10 cities versus a single city. We had previously identified a negative correlation between urbanization and genetic diversity across six sites within the city of Toronto (Rivkin and Johnson 2022). However, when we expanded our results to 10 cities, we did not detect a broad effect of urban habitat on genetic diversity. Instead, we identified microgeographic differences between cities that contributed to spatial patterns in genetic variation. We aligned our results to a reference genome instead of calling variants de novo and likely called a different set of SNPs in the current study, which may have contributed to these differences. Additionally, by including more sites and cities, we increased the power of our analyses to detect relatively small differences in genetic diversity and expanded the range of habitat variation in our analyses. This expansion of sites also allows us to distinguish patterns that are unique to a single urban landscape from those that reoccur across cities and reflect broadly acting urban evolutionary processes.

When we look to other systems, the effects of urbanization on genetic diversity vary substantially. A review of 194 urban genetic diversity studies identified a positive effect of urbanization on genetic diversity in 32% of cases and a negative effect in 62% of cases (Miles, Rivkin, et al. 2019). A separate review of mammal and bird genetic diversity found a negative effect of urbanization on genetic diversity in mammals but varied effects in birds (Schmidt et al. 2020). In plants, genetic diversity has been negatively and positively associated with urbanization depending on the system (Bartlewicz et al. 2015; Johnson et al. 2018; Rivkin and Johnson 2022; Caizergues et al. 2024), and in some cases urban populations act as reservoirs that harbor genetic diversity that maintains variation across the broader metapopulation (Roberts et al. 2007). Our results contribute to the growing body of evidence demonstrating the complex evolutionary responses of species to urbanization.

Although we found that genetic diversity was positively associated with impervious surface area, larger cities harbored less unique genetic variation than smaller cities. City area was negatively associated with the number of private alleles in a population, a trend primarily driven by Toronto (Figure 2C). Differences in gene flow among cities could contribute to variation in PA. Pollinators are the primary agents of gene flow in *I. capensis* , and pollinator services were maintained across an urbanization gradient in the nearby city of Ottawa (Barker and Sargent 2020). Large cities can harbor larger pollinator communities than small cities (Udy et al. 2020; Fauviau et al. 2024). For instance, larger cities may be more likely to have policies and funding available that promote pollinators such as reduced pesticides, restoration and connection of parks, and implementation of pollinator gardens. Toronto was the first “Bee City” in Canada and has invested considerable resources to restoring and connecting habitats for bees (Toronto's Pollinator Protection Strategy; livegreentoronto.ca). Toronto also has an extensive network of river valleys, which may contribute to increased admixture within the city and a reduction in genetic uniqueness across sites. However, we observed the most genetic structure in Toronto and high levels of admixture among the smaller cities, suggesting that Toronto sites may be the most genetically isolated in our sample.

An alternative explanation is that our results reflect the after‐effects of a population bottleneck. The number of unique alleles in a population may be lower following a bottleneck event or founder effect if the event results in the loss of rare alleles (Slatkin and Takahata 1985; Kalinowski 2004). This effect may be particularly noticeable in populations with lower rates of gene flow, such as those in Toronto. The current *N*_*e*_ of cities estimated by NeEstimator was lowest in Toronto (Table S1), suggesting that this city may not have fully recovered from the most recent bottleneck event, despite persistent gene flow across the region.

### 4.2 Demographic History

We identified repeated shifts in demographic history within the last 50 years in all cities. Results from the GONE analysis suggest that all cities experienced a severe population bottleneck that occurred between 10 and 50 years before sampling (Figure 4). We identified the same pattern when we grouped all cities together in a separate analysis, suggesting that the region may have experienced a single broad population crash (Figure S4). To our knowledge, our results constitute the first evidence of parallel population crashes in plants that has been documented across multiple cities. Two other recent studies have also identified recent population bottlenecks in the same region (Breitbart et al. 2025; Miles et al. 2025). Breitbart et al. (2025) found that in the city of Toronto and surrounding rural areas, the native plant common milkweed ( *Asclepias syriaca* ) has experienced a decline in *N*_*e*_ over the past 500 years, accelerating in the past five decades. Similarly, Miles et al. (2025) examined three native specialist herbivorous insects of milkweed across six cities in southern Ontario and found all three species experienced similar declines in *N*_*e*_ over the same time period. Interestingly, a common feature across all three studies is that the urban areas in the region have undergone rapid growth, with a 157% increase in built‐up area between 1971 and 2011 (Statistics Canada 2016). In contrast to our results, a global study of 24 cities that tested for parallel shifts in demographic history in white clover found that the species maintained higher *N*_*e*_ in urban than in rural habitats (Caizergues et al. 2024). These different responses to urbanization may reflect differences in habitat requirements and life history between these species, or the larger number of cities included in the study.

While our results are robust and logically follow from rapid land‐cover change in the region, some caveats warrant scrutiny when interpreting them. Although approximately half of the cities exhibited some level of recovery, current *N*_*e*_ values (generations 1–5) from GONE can be unreliable when dealing with reduced representation genotype data and high levels of admixture (Novo et al. 2023). Gene flow within cities may also upwardly skew GONE *N*_*e*_ estimates (Gargiulo et al. 2024), which likely contributes to the large maximum *N*_*e*_ values prior to the crashes. We observed substantially lower contemporary *N*_*e*_ values using NeEstimator, suggesting that most cities have not fully recovered from the recent bottleneck (Table S1). While the exact *N*_*e*_ from GONE should be interpreted with caution, the relative and consistent temporal changes reveal an important demographic response to rapid land‐use change in the recent past.

### 4.3 Gene Flow

Despite the evidence of recent bottlenecks in all cities, we observed a pattern of extensive gene flow across the region. At the broadest scale, most samples clustered together (Figure 2A). This trend may result from rapid urbanization, which facilitates gene flow by connecting populations through urban mosaics or human‐mediated transport. Our results are consistent with findings from other studies which have identified weak or non‐existent signatures of urbanization on plant genetic structure (Mollashahi et al. 2023; Ruas et al. 2024; Taichi et al. 2024). However, finer scaled reconstruction of population structure identified six clusters that were unrelated to city or habitat (Figure 2B). While Toronto largely formed two distinct clusters, most other cities were admixed. This result suggests that gene flow between cities has been fairly high, possibly signaling that pollination services for *I. capensis* are tolerant to the effects of urbanization (Barker and Sargent 2020). Our findings suggest that gene flow is likely a core contributor to the resilience of *I. capensis* to rapid urbanization.

Despite a strong signal of gene flow across the region, we detected population structure at the finest scales (i.e., within a city), where latitude, longitude, and microenvironmental variation all contributed to population differentiation (Figure 2C). Environmental variation in impervious surface area, precipitation and temperature was also associated with patterns in genetic diversity between sites, suggesting that these factors contributed to genetic differentiation. However, our MEMGENE model described only a small portion of the total genetic variation in our data, indicating that other factors contribute to genetic structure. For instance, differences in population census sizes among sites could impact the strength of genetic drift and alter patterns of population genetic differentiation (Wright 1931). Consistent with this hypothesis, Rivkin and Johnson (2022) identified an effect of population census size on genetic diversity that varied with urbanization in Toronto *I. capensis* populations. Additional factors such as variation in *N*_*e*_ and outcrossing rates likely also influence patterns of genetic structure within each city (Willi and Määttänen 2011).

### 4.4 Genetic Basis of Local Adaptation

City size was the strongest putative environmental driver of multilocus selection among populations, suggesting that variation in city size contributes to adaptive evolution in *I. capensis* . Our genome scans identified population structure as the strongest predictor of outlier loci (Figure 5), which is unsurprising given that we used a reduced‐representation sequencing approach (Hoban et al. 2016). After accounting for population structure, city area was the predictor most strongly associated with outliers, a pattern driven primarily by Toronto (Figure 5). Toronto is the second largest city in eastern North America and may impose stronger selection pressure on species than the smaller cities. Gene flow among populations limits the extent of local adaptation (Lenormand 2002), reducing the likelihood of detecting signatures of selection in the smaller cities that exhibited less genetic structuring than Toronto. We used a conservative approach to detect outlier loci by considering only loci shared across three independent approaches, thereby improving our chances of capturing true outliers (Forester et al. 2018). Most putatively adaptive loci that we identified were uncharacterized proteins, while several were associated with defensive functions. It is possible that increased herbivory or pathogen pressures in larger cities have led to selection on defensive genes if, for instance, city parks support higher abundances of herbivores than surrounding rural habitats that are closer to agricultural fields regularly sprayed with pesticides (Miles, Breitbart, et al. 2019; Rivkin and De Andrade 2023).

The genotype‐environment association (GEA) framework we used to assess the genetic basis of local adaptation in *I. capensis* was developed to detect adaptive genetic variation associated with environments (Rellstab et al. 2015; Frichot et al. 2013; Forester et al. 2018). GEA is a flexible suite of analyses that have been used to identify the genetic basis of local adaptation in managed and natural populations across a variety of environmental gradients and have been used to inform conservation and management in many contexts (Bernatchez et al. 2023). These methods can identify the same loci that show evidence of selection and local adaptation in reciprocal transplant experiments (Battlay et al. 2025). They are particularly well suited for species where it may be difficult or impractical to conduct common garden reciprocal transplant tests of local adaptation. Our analyses assessed environmental variation among cities using both local, population‐specific variables (impervious surface area, NDVI, temperature, and precipitation) and broader, city‐level variables (city area and growth rate). The inclusion of low‐resolution variables may have reduced our ability to detect loci under selection (Hoban et al. 2016). We identified loci that may facilitate adaptation in cities by increasing defense against herbivores and pathogens in urban parks. This finding would be supported by common garden reciprocal transplant experiments that explore specific defensive traits or identify defense genes to help uncover additional aspects of adaptation in *I. capensis* .

## 5 Conclusions

Our study examined patterns of neutral and adaptive genetic evolution in a species that can grow at high abundance in city greenspaces. We identified increased genetic diversity in more urban sites, despite evidence of repeated demographic declines across cities. Our findings demonstrate the complexity of evolutionary responses to urbanization and highlight the need for studies to consider multiple cities when assessing the influence of urbanization on patterns of genetic evolution. Parks and remnant natural habitats are important resources for many urban species. For *I. capensis* , and many other native species, parks are the primary habitat that allow for their survival in cities. These essential greenspaces facilitate eco‐evolutionary processes in many species and provide long‐term benefits for humans (Rivkin et al. 2019; Des Roches et al. 2020). Prioritizing the development and conservation of parks during city planning will contribute to the preservation and resilience of urban biodiversity.

## Funding

L.R.R. was funded by an NSERC Postdoctoral Research Fellowship and Queen Elizabeth II Scholarship. M.T.J.J. was funded by a Canada Research Chair, Steacie Fellowship, and NSERC Discovery Grant.

## Conflicts of Interest

The authors declare no conflicts of interest.

## Supporting information


**Table S2:** Results from linear models of genetic diversity estimated from 53 *Impatiens capensis* sites associated with environmental variation. Each model column section presents the slope and *p*‐value associated with the effect of the predictors on nucleotide diversity, observed heterozygosity, and the number of private alleles per population. Predictors include impervious surface area (ISA), normalized difference vegetation index (NDVI), mean annual temperature and precipitation, the area of the city (log‐transformed), and the human population growth rate of each city between 2016 and 2021. *p*‐values in bold denote significant effects (*p* < 0.05).
**Table S3:** City pairwise differentiation (*F*_ST_) for *Impatiens capensis* sites in 10 cities in southern Ontario. All cities were significantly differentiated (*p* < 0.05).
**Figure S1:** Map of the study area with a Principal Component Analysis of environmental variation across the region. (A) Variation in Principal Component (PC1), corresponding to increased impervious surface area (ISA) and temperature and decreased vegetation and precipitation. (B) Variation in PC2, corresponding to increased ISA, precipitation, and vegetation, and decreased temperature. Cities have been color coded.
**Figure S2:** Cross‐entropy scores from the sNMF analysis estimated from 10 replicates with 95% confidence intervals. Lower values denote a better model fit. Cross‐entropy scores plateau at *K* = 6, suggesting this value is optimal number of clusters.
**Figure S3:** Visualization of MEMGENE axes 1–3, explaining 38.1%, 22.8%, and 21.5% of shared genetic variation between individuals. Shapes denote urban (triangle) and rural (circle) sites, colors denote positive (blue) and negative (pink) eigenvectors, and the size of the shape is proportional to the magnitude of eigenvectors. Shapes that are a similar color and size represent individuals with shared genetic variation.
**Figure S4:** GONE estimates of recent demographic history of *Impatiens capensis* within the last 200 years (1 year = 1 generation for *I. capensis* ). All samples have been included as a single population. The bolded blue line represents the median *N*_*e*_ estimated across 100 replicates, and the shaded blue area represent the 95% confidence intervals from the runs.
**Figure S5:** Ordination plots of (A) RDA axes 1 (RDA1) and 2 (RDA2) and (B) RDA1 and RDA3. The small gray points at the center of the plot represent the SNPs, while the colored points represent individual samples color coded by city. The vectors show the environmental predictors included in the RDA. PC1 is the first principal component axis included from the genetic structure PCA to account for genetic structure in the model. Both plots are scaled symmetrically by the square root of the eigenvalues.

**Table S1:** Summary of environment and population genetic statistics from 53 *Impatiens capensis* sites.

**Table S4:** List of shared outlier loci identified, with their locations on each contig and associated BlastX hits and percent of query coverage.

## Data Availability

The data that support the findings of this study are openly available in NCBI at https://www.ncbi.nlm.nih.gov/sra/PRJNA1216546, reference number PRJNA1216546. R code and scripts can be found at https://github.com/ruthrivkin/Impatiens‐multicity‐evolution.
