# Supplementary material for: A Multi‐City Assessment of Genomic Evolution in the Native Wildflower Impatiens capensis
Source: Evol Appl. 2026 Apr 22;19(4):e70218. doi: 10.1111/eva.70218 (PMC13103269; doi:10.1111/eva.70218)
Supplement: Supplementary file 1 — Table S2: Results from linear models of genetic diversity estimated from 53 Impatiens capensis sites associated with environmental variation. Each model column section presents the slope and p‐value associated with the effect of the predictors on nucleotide diversity, observed heterozygosity, and the number of private alleles per population. Predictors include impervious surface area (ISA), normalized difference vegetation index (NDVI), mean annual temperature and precipitation, the area of the city (log‐transformed), and the human population growth rate of each city between 2016 and 2021. p‐values in bold denote significant effects (p < 0.05). Table S3: City pairwise differentiation (F ST) for Impatiens capensis sites in 10 cities in southern Ontario. All cities were significantly differentiated (p < 0.05). Figure S1: Map of the study area with a Principal Component Analysis of environmental variation across the region. (A) Variation in Principal Component (PC1), corresponding to increased impervious surface area (ISA) and temperature and decreased vegetation and precipitation. (B) Variation in PC2, corresponding to increased ISA, precipitation, and vegetation, and decreased temperature. Cities have been color coded. Figure S2: Cross‐entropy scores from the sNMF analysis estimated from 10 replicates with 95% confidence intervals. Lower values denote a better model fit. Cross‐entropy scores plateau at K = 6, suggesting this value is optimal number of clusters. Figure S3: Visualization of MEMGENE axes 1–3, explaining 38.1%, 22.8%, and 21.5% of shared genetic variation between individuals. Shapes denote urban (triangle) and rural (circle) sites, colors denote positive (blue) and negative (pink) eigenvectors, and the size of the shape is proportional to the magnitude of eigenvectors. Shapes that are a similar color and size represent individuals with shared genetic variation. Figure S4: GONE estimates of recent demographic history of Impatiens capensis within the last [file EVA-19-e70218-s003.docx]

**Supplemental Material**

**A multi-city assessment of genomic evolution in the native wildflower *Impatiens capensis***

L. Ruth Rivkin^1,2,3,4,*^, Colin J Garroway^1^, Marc T.J. Johnson^5^

^1^Department of Biological Sciences, University of Manitoba, Winnipeg, Manitoba, Canada; ^2^Polar Bears International, Bozeman, Montana, USA; ^3^San Diego Zoo Wildlife Alliance, Escondido, California, USA; ^4^University of Toronto, Toronto, Ontario, Canada; ^5^University of Toronto Mississauga, Mississauga, Ontario, Canada

*Author for correspondence: [ruth.rivkin@umanitoba.ca](mailto:ruth.rivkin@umanitoba.ca)

**Author details**

LRR: [ruth.rivkin@umanitoba.ca](mailto:ruth.rivkin@umanitoba.ca), ORCID: 0000-0003-2632-3388

CCG: [colin.garroway@umanitoba.ca](mailto:colin.garroway@umanitoba.ca), ORCID: 0000-0002-0955-0688

MTJJ: [marc.johnson@utoronto.ca](mailto:marc.johnson@utoronto.ca), ORCID: 0000-0001-9719-0522

**Data Availability Statement**

The data that support the findings of this study are openly available in NCBI at <https://www.ncbi.nlm.nih.gov/sra/PRJNA1216546>. R code and scripts can be found at <https://github.com/ruthrivkin/Impatiens-multicity-evolution>.

Table S1. Summary of environment and population genetic statistics from 53 *Impatiens capensis* sites. CSV file attached.

Table S2. Results from linear models of genetic diversity estimated from 53 *Impatiens capensis* sites associated with environmental variation. Each model column section presents the slope and p-value associated with the effect of the predictors on nucleotide diversity, observed heterozygosity, and the number of private alleles per population. Predictors include impervious surface area (ISA), normalized difference vegetation index (NDVI), mean annual temperature and precipitation, the area of the city (log-transformed), and the human population growth rate of each city between 2016-2021. P-values in bold denote significant effects (p < 0.05).

|  |  | Nucleotide Diversity | | Observed heterozygosity (H_O_) | | Private alleles (PA) | |
| --- | --- | --- | --- | --- | --- | --- | --- |
| Predictors | DF | slope (*b*) | p-value | slope (*b*) | p-value | slope (*b*) | p-value |
| ISA | 1 | 4.68E-03 | **0.008** | 0.03 | **0.021** | -273.69 | 0.174 |
| NDVI | 1 | 6.61E-03 | 0.071 | 0.05 | 0.085 | -362.15 | 0.388 |
| Temperature | 1 | -8.71E-05 | 0.782 | -1.65E-03 | 0.514 | 100.91 | **0.008** |
| Precipitation | 1 | -4.45E-06 | 0.424 | -5.88E-05 | 0.189 | 1.31 | **0.048** |
| log(City area) | 1 | -5.50E-05 | 0.720 | 2.03E-03 | 0.103 | -41.49 | **0.024** |
| Population growth rate | 1 | -1.52E-05 | 0.691 | -1.86E-04 | 0.543 | 1.05 | 0.814 |

Table S3. City pairwise differentiation (F_ST_) for *Impatiens capensis* sites in 10 cities in southern Ontario. All cities were significantly differentiated (p < 0.05).

|  | Brantford | Georgetown | | Guelph | Burlington | | Cambridge | | Orangeville | | Hamilton | Kitchener | Milton |
| --- | --- | --- | --- | --- | --- | --- | --- | --- | --- | --- | --- | --- | --- |
| Georgetown | 0.02 | |  |  | |  | |  | |  |  |  |  |
| Guelph | 0.02 | | 0.02 |  | |  | |  | |  |  |  |  |
| Burlington | 0.02 | | 0.02 | 0.02 | |  | |  | |  |  |  |  |
| Cambridge | 0.02 | | 0.03 | 0.03 | | 0.02 | |  | |  |  |  |  |
| Orangeville | 0.03 | | 0.02 | 0.02 | | 0.02 | | 0.02 | |  |  |  |  |
| Hamilton | 0.03 | | 0.04 | 0.04 | | 0.03 | | 0.03 | | 0.04 |  |  |  |
| Kitchener | 0.01 | | 0.02 | 0.01 | | 0.01 | | 0.02 | | 0.01 | 0.03 |  |  |
| Milton | 0.03 | | 0.02 | 0.03 | | 0.02 | | 0.03 | | 0.03 | 0.04 | 0.02 |  |
| Toronto | 0.03 | | 0.02 | 0.03 | | 0.02 | | 0.03 | | 0.02 | 0.04 | 0.02 | 0.03 |

Table S4. List of shared outlier loci identified, with their locations on each contig and associated BlastX hits and percent of query coverage. XLSX file attached.


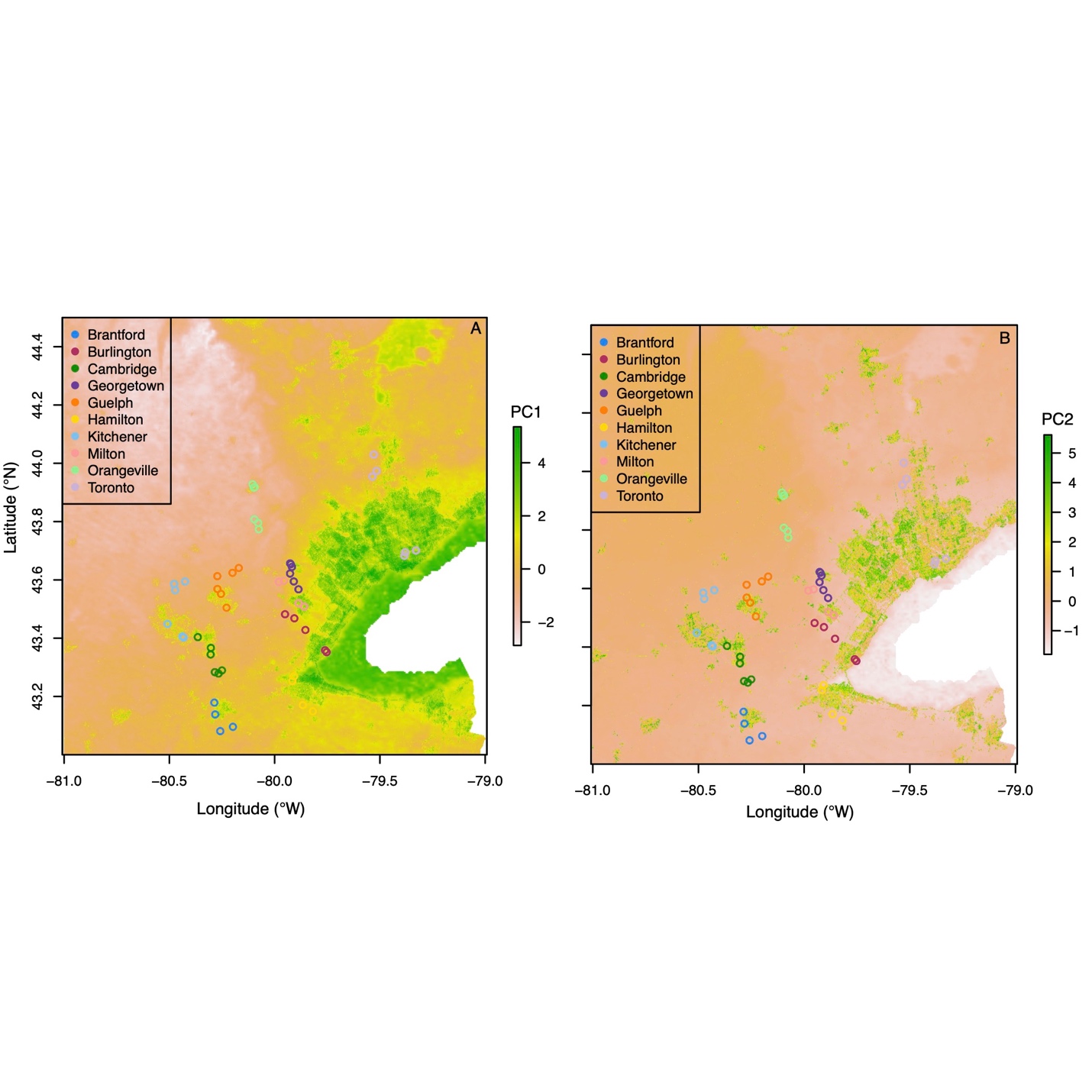
Figure S1. Map of the study area with a Principal Component Analysis of environmental variation across the region. A) Variation in Principal Component (PC1), corresponding to increased impervious surface area (ISA) and temperature and decreased vegetation and precipitation. B) Variation in PC2, corresponding to increased ISA, precipitation, and vegetation, and decreased temperature. Cities have been color coded.

Figure S2. Cross-entropy scores from the sNMF analysis estimated from 10 replicates with 95% confidence intervals. Lower values denote a better model fit. Cross-entropy scores plateau at K = 6, suggesting this value is optimal number of clusters.


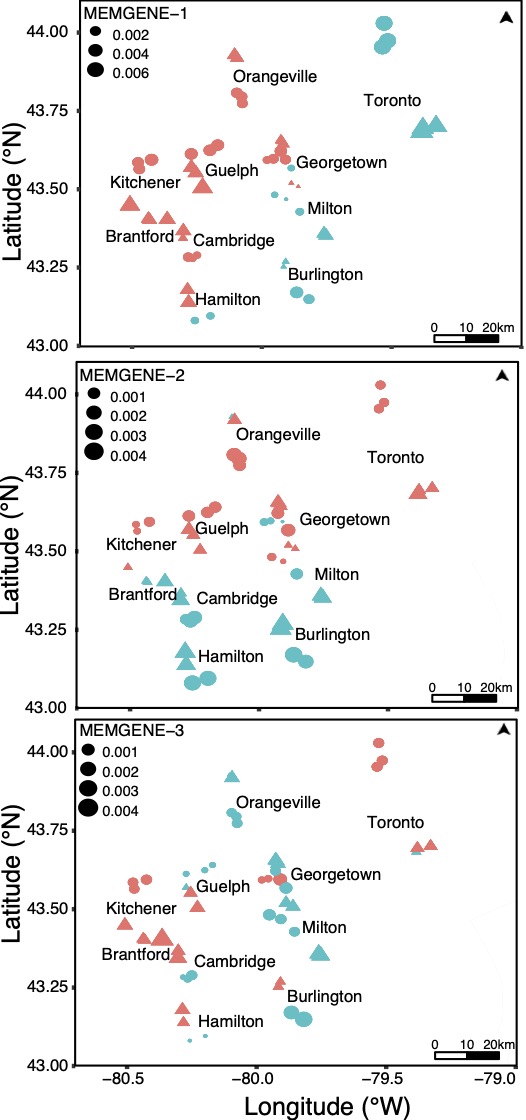


Figure S3. Visualization of MEMGENE axes 1-3, explaining 38.1%, 22.8%, and 21.5% of shared genetic variation between individuals. Shapes denote urban (triangle) and rural (circle) sites, colors denote positive (blue) and negative (pink) eigenvectors, and the size of the shape is proportional to the magnitude of eigenvectors. Shapes that are a similar color and size represent individuals with shared genetic variation.

Figure S4. GONE estimates of recent demographic history of *I. capensis* within the last 200 years (1 year = 1 generation for *I. capensis*). All samples have been included as a single population. The bolded blue line represents the median *N_e_* estimated across 100 replicates, and the shaded blue area represent the 95% confidence intervals from the runs.

Figure S5. Ordination plots of A) RDA axes 1 (RDA1) and 2 (RDA2) and B) RDA1 and RDA3. The small grey points at the centre of the plot represent the SNPs, while the coloured points represent individual samples color coded by city. The vectors show the environmental predictors included in the RDA. PC1 is the first principal component axis included from the genetic structure PCA to account for genetic structure in the model. Both plots are scaled symmetrically by the square root of the eigenvalues.
